# Antibody-Dependent Cytotoxicity of Monocytes in Preeclampsia Is Associated with Soluble Forms of HLA

**DOI:** 10.3390/ijms262311638

**Published:** 2025-12-01

**Authors:** Aleksey M. Krasnyi, Leya E. Sorokina, Anastacia Maria Argentova-Stevens, Diana N. Kokoeva, Aleksey A. Alekseev, Tatjana Jankevic, Natalia E. Kan, Victor L. Tyutyunnik, Gennady T. Sukhikh

**Affiliations:** 1 National Medical Research Center for Obstetrics, Gynecology and Perinatology Named after Academician V.I. Kulakov of Ministry of Healthcare of Russian Federation, 117997 Moscow, Russia; 2 DNA-Technology LLC, 117105 Moscow, Russia

**Keywords:** preeclampsia, monocytes, antibody-dependent cytotoxicity, CD16, HLA, HLA class II, HLA class I, DRB1*01:01:01G

## Abstract

Preeclampsia (PE) is a serious gestational complication that affects the lives of the mother and the child. Women with PE showed higher levels of pro-inflammatory cytokines secreted by leukocytes compared with women with normal pregnancies. The differences are most noticeable in the percentage of CD16+ monocytes, although the mechanism underlying this increase remains unclear. The CD16 receptor is critical for antibody-dependent cellular cytotoxicity, and by binding to antibodies on the surface of target cells, it activates their death. In this study, we examined the effect of soluble placental factors on the expression of CD16 monocytes and the potential role the soluble form of human leukocyte antigen (HLA) has on CD16 monocyte expression. At the first stage of our study, we collected samples of placental villi fragments from 58 pregnant women (38 women with PE and 20 with a healthy pregnancy). Then we studied the effect of placental villus-conditioned culture medium on CD16 expression by monocytes derived from the same women. It was shown that the content of CD16+ monocytes increased significantly in women with PE within 3 h and to a lesser extent in women with a healthy pregnancy (*p* = 0.009). Also, the addition of the recombinant histocompatibility HLA-B to the placental villus-conditioned culture medium blocks the induction of CD16 expression on monocytes. At the second stage of our study, we typed HLA class I and class II alleles in the umbilical cord blood samples and the venous blood samples taken from 38 women with PE and 40 women with a normal pregnancy. It was found that certain HLA class II alleles predominate in women with preeclampsia. The DRB1*01:01:01G allele showed the greatest difference (*p* < 0.001). Analyzing five alleles simultaneously makes it possible to predict the PE with AUC = 0.76. Evaluation of unique children’s alleles also showed that class II alleles have greater differences among them than class I alleles. The DQB1*06:03:01G allele had the greatest differences with *p* = 0.03 (the number was higher in the control group). Performing an analysis of four alleles of children simultaneously allowed us to predict PE with an AUC of 0.64. This work suggests that the activation of CD16+ monocyte expression occurs due to the interaction of soluble placental antigens with monocytes. The most likely way to activate CD16 expression on monocytes is by HLA class II (both maternal and fetal) interaction with CD4 receptors on the surface of monocytes, whereas HLA class I is capable of blocking this process. Evaluation of maternal HLA alleles may be a significant marker for predicting PE.

## 1. Introduction

It is estimated that about 3–5% of all pregnancies are complicated by preeclampsia (PE), which causes at least 42,000 maternal deaths annually [1]. The mechanism of PE development is not clear, and various factors can cause this condition. In general, PE is characterized by activation of endothelial cells, intravascular inflammation, and syncytiotrophoblast stress [2]. One of the mechanisms of PE development is the immune rejection of a genetically different fetus, since normal placentation requires the development of immune tolerance between the fetus and the mother [3]. In the case of pregnancies through oocyte donation (OD) with an allogeneic fetus, there is an increased risk of the maternal immunoregulation mechanisms failing to adapt to the embryo [4]. The prevalence of PE is reported to be higher for such pregnancies [4,5]. The mechanisms of transplant rejection have been highly studied, which indicates that monocytes and macrophages play a key role in transplant rejection, since these mononuclear phagocytic cells are capable of recognizing alloantigens [6]. This mechanism is not exactly clear. According to some data, monocytes can directly interact with soluble forms of HLA. Thus, it was shown that the activation of the monocyte surface CD4 glycoprotein through interaction with the soluble form of the HLA class II can lead to the expression of various cell surface markers, including CD16 [7]. However, the authors could not find any effect of HLA class I on monocytes. According to some data, the soluble form of the HLA class I can cause suppression of the immune response during organ transplantation [8]. One of the signs of transplant rejection is an increasing amount of CD16+ monocytes [9,10]. The CD16 receptor, as a low-affinity IgG receptor, is crucial for antibody-dependent cellular cytotoxicity (ADCC), and when binding to antibodies on the surface of target cells, it activates their death. In contrast, CD16− monocytes do not exhibit ADCC [11]. As mentioned above, PE exhibits processes similar to the immune response during transplant rejection. PE has been shown to significantly increase the percentage of CD16+ monocytes in peripheral blood [12]. The connection between the percentage of CD16+ monocytes in peripheral blood and the severity of PE has also been established [13]. The mechanisms of increased CD16 expression on monocytes during pregnancy are not clear.

This work aims to study the potential mechanism that induces CD16 expression on monocytes during PE. To achieve this, we studied how placental villus-conditioned culture medium affects CD16 expression on the monocytes of women in PE and studied the effect of HLA-B as a potential blocker of this process. To confirm the role of HLA in PE development, as well as to identify the potential use in diagnostics, we typed HLA class I and class II alleles in samples taken from the umbilical cord blood and maternal blood.

## 2. Results

### 2.1. Clinical Characteristics of Patients

According to the study design, two groups were initially formed at the first stage: women with a physiological course of pregnancy (*n* = 20) and pregnant women with diagnosed preeclampsia (*n* = 38). Among the latter, ten women had severe preeclampsia and twenty-eight had a moderate form. At the second stage, an additional twenty healthy pregnant women were included in the study. All the patients included in this study were comparable in terms of social status, bad habits, and occupational hazards. The main anamnestic and clinical data of the analyzed groups are presented in Table 1.

The age range of the women in our research was from 18 to 45 years. The age for women with PE was 33.0 (29.5; 34.5) years, and for women with healthy pregnancies it was 30.5 (29.0; 34.0) years (*p* = 0.098). There was no statistically significant difference in body mass index between the groups: 29.0 (24.0; 32.0) kg/m^2^ for the PE group versus 25.5 (23.7; 28.2) kg/m^2^ for the control group (*p* = 0.062).

As expected, significant intergroup differences were revealed in blood pressure (*p* = 0.001), urine protein concentration (*p* < 0.001), and the frequency of edema syndrome (*p* = 0.01), which are traditionally considered the primary criteria for the diagnosis of preeclampsia. We considered anamnestic risk factors for the development of preeclampsia, among which statistically significant differences were obtained only for chronic arterial hypertension, which was significantly more common in patients with preeclampsia (*p* < 0.001). The obtained differences in the frequency of chronic arterial hypertension between the preeclampsia and control groups are consistent with literature data, which show that the risk of developing preeclampsia in women with chronic hypertension reaches 13–40% [14]. The question of the relationship between chronic hypertension and preeclampsia remains incompletely understood. Meanwhile, it is known that both hypertension and preeclampsia affect the condition of the arteries with the development of endothelial dysfunction [15]. The occurrence of other somatic diseases, including vascular pathologies, kidney diseases, and diabetes, did not have statistically significant differences between the groups (*p* > 0.05). The average gestational age at delivery in the PE group was 35.7 (31.0; 39.0) weeks, while in the control group it was 39.0 (39.0; 40.0) weeks (*p* < 0.001). There was a higher number of patients with PE who had to undergo a surgical delivery: in 50% of cases, a cesarean section was performed as an emergency (*p* < 0.001), and in 34.2%, it was planned (*p* = 0.005). In addition, children born to women with complicated pregnancies had a significantly lower body weight-2265.5 (1420.0; 3390.0) g compared with newborns in the control group-3367.0 (3142.5; 3685.0) g (*p* < 0.001). Women with PE were more likely to be diagnosed with fetal growth retardation (*p* = 0.002).

### 2.2. The Effect of Placental Soluble Factors on CD16 Expression by Monocytes

**Figure 1:**
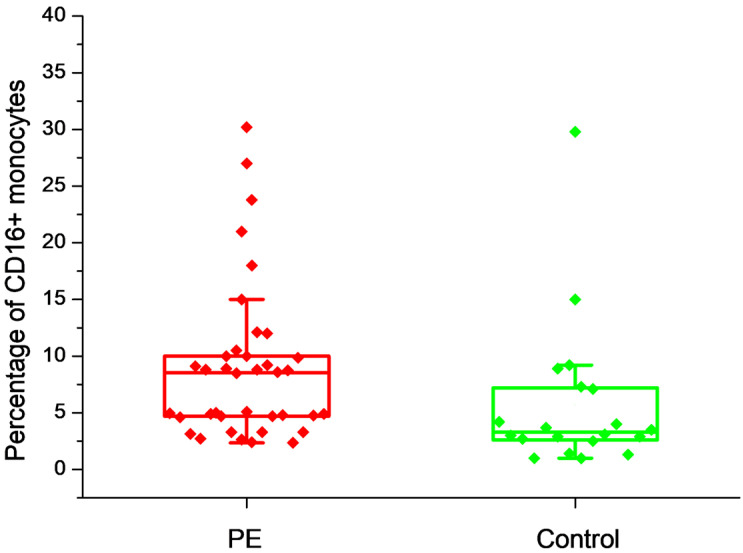
The percentage of CD16+ monocytes 24 h after delivery is shown in the group of patients with PE and in the control group.

To assess the ability of soluble placental antigens to activate CD16 expression, we obtained a fragment of the villous part of the placenta from each patient and placed it in a culture medium for 24 h. After that, we studied the effect of placental villus-conditioned culture medium (PVCM) on the CD16 expression by monocytes obtained from the same women the day after childbirth. The adhesive method was used to isolate monocytes from peripheral blood mononuclear cells (PBMC). The cells were exposed to PVCM in 24-well plates for 3 h. After that, the percentage of CD16+ monocytes was determined. In the control wells, we used a culture medium. Figure 1 shows the CD16+ monocytes content 24 h after delivery in the control wells. In the samples obtained from patients with PE, the percentage of CD16+ monocytes was statistically significantly higher, *p* = 0.005. Control wells with monocytes obtained from women with healthy pregnancies showed 3.3 (2.6; 7.1)%, and in wells with monocytes obtained from women with PE, it was 8.5 (4.7; 10)%.

**Figure 2:**
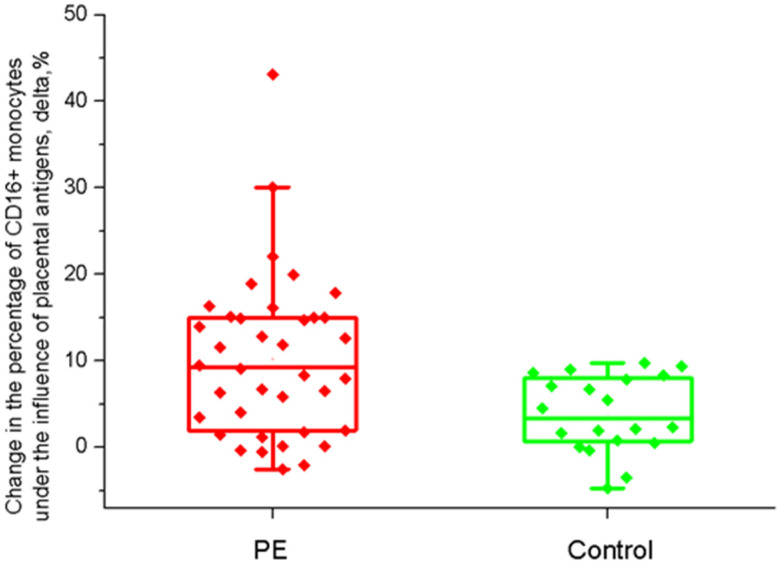
The effect of soluble placental factors on maternal CD16+ monocytes. Placenta and blood were obtained from the same patient. The delta value represents the difference between the percentage in control wells and in wells with placental villus-conditioned culture medium; both groups of wells contain monocytes from the same women.

Figure 2 shows the effect of soluble placental factors on maternal monocytes. The data are presented as the difference between the results in the control well and in the well with PVCM (delta). The delta value was statistically significantly higher in patients with PE and amounted to 9.3 (2.3; 15)%, while only showing 3.4 (0.7; 7.9)% in the control group (*p* = 0.009).

When comparing severe and moderate PE, the delta value was higher for severe PE and resulted in 12.1 (7.3; 18.2)%, while being only 8.1 (1.3; 14.9)% for moderate PE. The difference was close to being statistically significant (*p* = 0.07). When conducting a correlation analysis between the percentage of CD16+ monocytes in the blood 24 h after delivery and the delta value, we found a connection both in the PE group and in the control group. The Spearman coefficient values were 0.99, *p* < 0.001 in both cases.

### 2.3. The Effect of HLA Class I (HLA-B) on CD16 Expression on Monocytes

**Figure 3:**
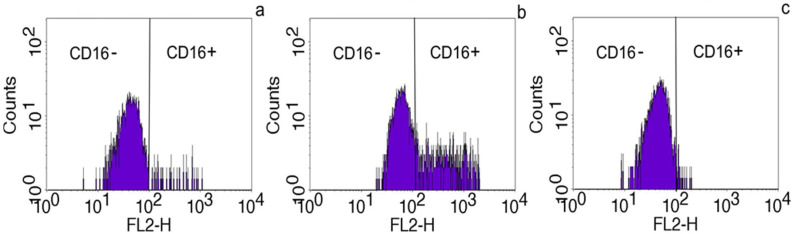
(**a**)—CD16+ monocytes obtained one day after delivery from a patient with PE; (**b**)—the same monocytes in the placental villus-conditioned culture medium (PVCM) for 3 h; (**c**)—the same monocytes in the PVCM and the recombinant histocompatibility antigen HLA-B for 3 h.

To demonstrate that HLA class I can exhibit immune suppression through inhibiting CD16+ monocyte expression, we conducted the following experiment. Monocytes were obtained one day after delivery from a patient who developed PE and were placed in three wells of a 24-well plate. In the control well, only medium was added, while the second well had PVCM. The third well contained PVCM and recombinant histocompatibility antigen HLA-B. After 3 h, the level of CD16+ monocytes was examined in each well (Figure 3). It was established that the recombinant HLA-B completely blocked CD16 expression caused by PVCM. The experiment was repeated 10 times, and in all cases we observed complete suppression of CD16 expression.

Evaluation of class I and class II alleles in blood from the umbilical cord and from women with PE and women with healthy pregnancies. Alleles of class I and class II histocompatibility complexes were evaluated in blood samples from the umbilical cord of 38 women with PE and 40 women with healthy pregnancies. The complete HLA evaluation data are presented in the Supplementary File S1. Fisher’s exact test was used to verify the associations between certain maternal HLA alleles and the development of PE during pregnancy. Five maternal HLA class II alleles showed statistically significant differences (Table 2). The DRB1*01:01:01G allele showed the greatest differences (*p* < 0.001).

**Figure 4:**
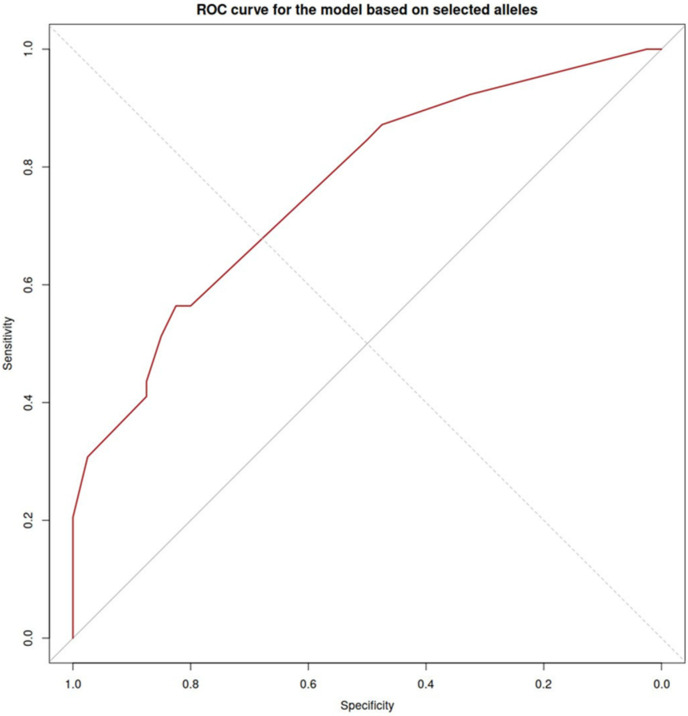
ROC curve to evaluate PE occurrence based on maternal HLA class II alleles.

The evaluation of the prognostic value of PE development during pregnancy using the five indicated alleles in Table 2 showed AUC = 0.76 (0.66–0.86). The ROC curve is shown in Figure 4.

To assess the effect of paternal HLA alleles on the development of PE, we evaluated HLA alleles in samples of the umbilical cord blood taken from women with PE. Beforehand, we excluded the alleles that duplicated the maternal ones. Four paternal HLA class II alleles showed statistically significant or close to significant differences (Table 3).

**Figure 5:**
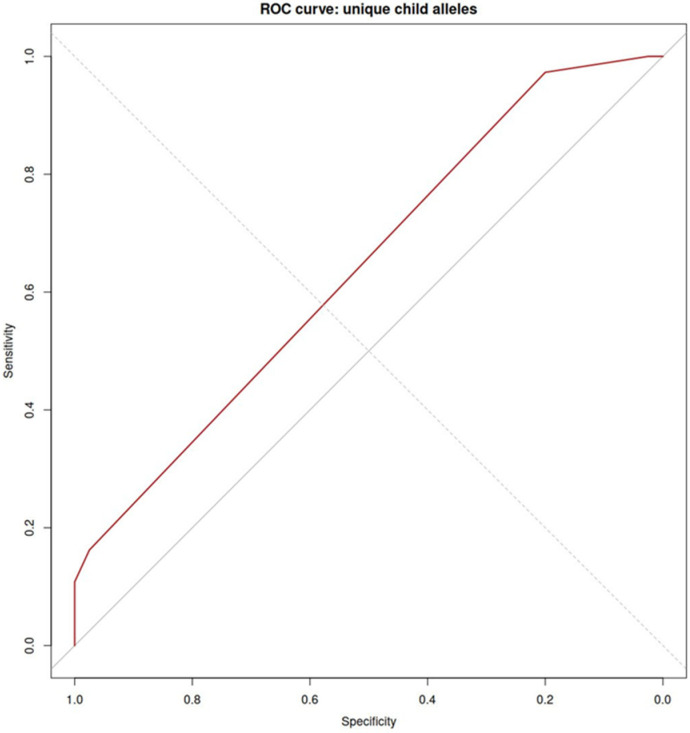
ROC curve for predicting PE based on unique child (paternal) HLA class II alleles.

The ROC curve based on the results of these alleles has an area under the curve equal to 0.64 (0.56–0.72) (Figure 5).

## 3. Discussion

**Figure 6:**
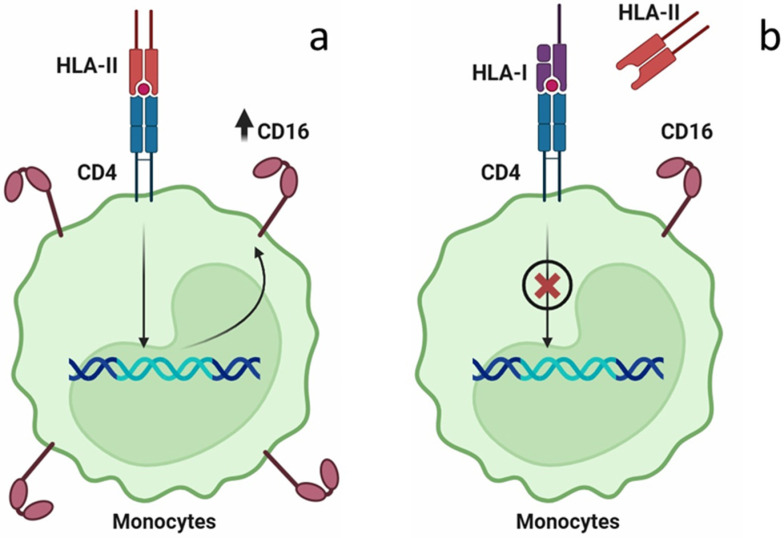
(**a**)—soluble form of HLA class II leads to the CD16 expression by monocytes. (**b**)—soluble form of HLA class I blocks this process by acting as the CD4 receptor antagonist.

Preeclampsia is widely known as a multifactorial disease involving immune dysregulation, impaired placentation, angiogenic imbalance, vascular injury, and maternal constitutional factors [16]. Early pregnancy is characterized by the establishment of immune tolerance to the semiallogeneic fetus. Disruptions in this process, including abnormal maternal–fetal HLA interactions, can provoke increased activation of innate immune cells. Previous studies have shown an association between certain HLA class II mismatches or maternal anti-fetal immune responses and the risk of preeclampsia, suggesting that altered antigen presentation and monocyte/macrophage activation may contribute to abnormal placentation [17]. Alongside immune mechanisms, impaired remodeling of the spiral arteries and syncytiotrophoblast stress lead to the release of anti-angiogenic factors, such as sFlt-1 and soluble endoglin, resulting in endothelial dysfunction, hypertension, and systemic inflammation [16,18]. Oxidative stress and microvascular damage amplify this response. Our findings fit into this broader pathophysiological framework: placental soluble antigens capable of enhancing CD16 expression on monocytes provide a plausible early pathway for immune system activation, contributing to systemic inflammation. The observed association between maternal HLA class II alleles and preeclampsia suggests that maternal genetic predisposition shapes the immune response to placental signals. Although these interactions represent just one component of the multifactorial disease process, they highlight a mechanistic link between genetic background and functional immunological activation. A critical manifestation of this immune dysregulation is the observed increase in non-classical and intermediate monocytes, characterized by CD16 (FcγRIII) expression, in women with preeclampsia [12,13]. Notably, the level of CD16+ monocytes has been shown to correlate with disease severity [13], positioning these cells as a key contributor to the pathogenesis of PE. Given that CD16 is instrumental in antibody-dependent cellular functions and pro-inflammatory responses, this study aimed to elucidate the mechanism by which its expression on monocytes is induced during pregnancy, potentially in response to placental signals. It is known that one of the mechanisms of CD16 expression by monocytes is the interaction of the CD4 receptor with the soluble form of HLA class II [7]. In our work, we have demonstrated that soluble placental factors are able to induce CD16 expression by monocytes. We assumed that this occurs due to the interaction with placental HLA class II. To confirm this, we conducted an experiment to block this process by adding a soluble form of HLA Class I, which is known to have an immunosuppressive effect [8]. Our assumption was confirmed, which allowed us to conclude that HLA class II acts as an agonist and HLA class I as an antagonist when interacting with CD4 receptors of monocytes (Figure 6). Although CD4 receptor involvement was not directly tested in this study, our results are consistent with the previously described mechanism [7]. In this study, we investigated the immunosuppressive effect of HLA-B, a subtype of HLA-I, on monocytes. Our results leave it unclear whether other HLA-I subtypes possess similar properties, a question that warrants further investigation.

One of the potential factors that can neutralize the immune activity of HLA are antibodies directed against them. However, their association with pregnancy outcomes is not straightforward, as a meta-analysis conducted by Lashley et al. did not reveal a consistent effect of antibodies against HLA class I or II on pregnancy outcome [19]. We established that the level of CD16+ monocytes in pregnant women correlated with the ability of placental factors to induce CD16 expression by monocytes. This suggests that placental factors, namely the soluble forms of HLA, determine the expression of CD16 by monocytes. To find which forms may be associated with PE, we evaluated alleles of HLA class I and class II from the umbilical cord blood and maternal blood. We expected that paternal antigens would play the main role, but regression analysis showed that maternal HLA has the greatest predictive value. Assessing the presence of only 5 HLA class II maternal alleles allowed us to predict PE with good prognostic value (AUC = 0.76), whereas paternal HLA class II AUC was 0.64. The obtained results demonstrate the potential for predicting preeclampsia by combining HLA gene alleles and blood markers. Further optimization of the model and multivariate integration may lead to high-precision prediction of the complication. In this work, we demonstrated that the cytotoxicity of monocytes can be regulated by soluble HLA molecules. This result may have practical applications, such as when cytotoxicity needs to be reduced (preeclampsia, organ transplantation) or increased (cancer). It should be noted, however, that this study has several limitations. The limited sample size, insufficient multi-ethnic and multi-regional data, and the lack of long-term prognosis assessment are issues that should be addressed in future research.

## 4. Materials and Methods

Patients. We conducted a case–control study of two groups: patients with a healthy occurring pregnancy (*n* = 40) and women experiencing PE during pregnancy (*n* = 38). Patients with severe extragenital pathologies (diabetes, chronic kidney disease, autoimmune diseases etc.), multiple pregnancies, fetal malformations, genetic diseases of the mother and fetus, acute or chronic infectious diseases etc., were excluded from the study.

PVCM effect on CD16 expression by monocytes. At the first stage of our research, we studied the effect of placental villus-conditioned culture medium (PVCM) on CD16 expression by monocytes and the potential role of the HLA soluble form in CD16 monocyte expression. All 38 patients with preeclampsia and 20 with healthy pregnancies were examined. A fragment of the placental villous part weighing 0.12–0.15 g was obtained from each patient, washed with sterile phosphate-buffered saline (PBS) to remove blood, and placed in 3 mL of ImmunoCult medium™-XF T Cell Expansion Medium (ICM) (StemCell Technologies, Vancouver, BC, Canada) for 24 h in a CO_2_ incubator to produce PVCM. Blood samples taken with the anticoagulant heparin were obtained the day after delivery. Peripheral blood mononuclear cells (PBMCs) were extracted from blood using the standard method. Next, PBMCs from each patient were placed in two wells of a 24-well plate with ICM, and after 30 min they were washed with a medium to remove lymphocytes that did not become attached to the bottom of the well. Monocytes were isolated the day after delivery so that they still had maximum viability for the experiment. Next, the medium in one of the wells was replaced on PVCM and left in a CO2 incubator for 3 h. After that, the number of CD16+ monocytes in both wells was determined. Monoclonal antibodies CD16 (Biolegend, San Diego, CA, USA) were used for immunophenotyping. A monocyte gate was identified based on side scatter (SSC) and CD45 expression (BD Biosciences Systems & Reagents, San Jose, CA, USA). BD FACSCalibur flow cytometer with CellQuest Pro software version 5.2. (BD Biosciences Systems & Reagents, San Jose, CA, USA) was used for the analysis.

HLA-B effect on CD16 monocyte expression. To study the effect of HLA-I (HLA-B) on CD16 monocyte expression, monocytes obtained one day after delivery from a patient with PE were placed in three wells of a 24-well plate. The control well contained only ICM, and the second well had PVCM. The third well contained PVCM and recombinant human HLA class I histocompatibility antigen, B alpha chain (HLA-B), partial, Code CSB-YP355776HU (Cusabio Biotech, Wuhan, China) at a concentration of 10 μg/mL. After 3 h, the level of CD16+ monocytes was examined in each well.

HLA typing. HLA typing was performed using the Illumina MiSeq platform (San Diego, CA, USA) and the TruSight HLA v2 kit (Illumina, San Diego, CA, USA). All samples were processed manually according to the Illumina procedure.

Statistical Analysis. The significance of the differences between the groups was analyzed using the two-sided Mann–Whitney U test. The results are presented as the median, upper, and lower quartiles (Me (Q1; Q3)). Spearman’s correlation coefficient (rs) was applied to study the connection between the studied factors. Categorical variables were analyzed using Fisher’s exact test. Differences were considered statistically significant at *p* < 0.05. SPSS Statistics 27 (IBM, Armonk, NY, USA) and OriginPro 8.5 (OriginLab Corporation, Northampton, MA, USA) programs were used for the statistical processing of the results and plotting graphs. The bioinformatic analysis performed to evaluate HLA alleles is described in the Supplementary File S2.

## 5. Conclusions

We have shown that CD16 expression by monocytes is activated directly by the interaction with soluble placental antigens. The most likely way to activate CD16 expression by monocytes is through the interaction of HLA class II (both maternal and fetal) with CD4 receptors on the surface of monocytes, whereas HLA class I blocks this process. Genotyping of maternal HLA alleles may be used for diagnosing PE. These results may have practical significance, not only for PE but also for conditions caused by monocyte-mediated cytotoxicity.

## Figures and Tables

**Table 1:** Anamnestic and clinical data of pregnant women.

Characteristics	Women with PE (*n* = 38)	Healthy Women (*n* = 40)	*p*-Value
Age, years	33.0 (29.5; 34.5)	30.5 (29.0; 34.0)	0.098
BMI, kg/m^2^	29.0 (24.0; 32.0)	25.5 (23.7; 28.2)	0.062
Primigravida	17 (43.6)	17 (42.5)	0.922
Primipara	20 (51.3)	20 (50.0)	0.909
Preeclampsia in the anamnesis	5 (12.8)	0	0.026
Systolic blood pressure, mm Hg	145.0 (140.0; 153.5)	115.0 (110.1; 120.5)	0.001
Diastolic blood pressure, mm Hg	90 (86; 100)	70 (60; 75)	0.001
Protein in urine, g/L	0.77 (0.30; 1.65)	0.05 (0.03; 0.12)	<0.001
Edema	17 (43.6)	6 (15.0)	0.01
Chronic arterial hypertension	12 (30.8)	1 (2.5)	<0.001
Chronic liver disease	12 (30.8)	6 (15.0)	0.093
Vascular disease	4 (10.3)	6 (15.0)	0.737
Diabetes	1 (2.6)	0	0.494
Gestational age at delivery, weeks	35.7 (31.0; 39.0)	39.0 (39.0; 40.0)	<0.001
Cesarean section			
– emergency	19 (50.0)	6 (15.0)	<0.001
– planned	13 (34.2)	13 (34.2)	0.005
Newborn weight, g	2265.5 (1420.0; 3390.0)	3367.0 (3142.5; 3685.0)	<0.001
Fetal growth restriction	8 (21.1)	0	0.002

Data are presented as an absolute number and proportion (%) of patients, Fisher’s exact criterion. Me (Q1; Q3) data are presented as a median with an interquartile interval (Mann–Whitney test). BMI—body mass index.

**Table 2:** Maternal alleles that showed statistically significant differences in PE.

Allele	Preeclampsia (*n* = 38)		Control (*n* = 40)		*p* Value
	*n*	F (%)	*n*	F (%)	
DRB1*01:01:01G	12	30.8	1	2.5	<0.001
DQB1*05:01:01G	13	33.3	4	10	0.014
DPB1*04:01:01G	22	56.4	33	82.5	0.015
DRB4*01:01:01G	11	28.2	22	55	0.022
DQA1*03:01:01G	5	12.8	14	35	0.034

F—frequency.

**Table 3:** Paternal alleles showing statistically significant differences in PE.

Allele	Preeclampsia (*n* = 36)		Control (*n* = 40)		*p* Value
	n	F (%)	n	F (%)	
DQB1*06:03:01G	1	2.8	8	20	0.029
DRB1*04:01:01G	4	11.1	0	0	0.049
DQB1*03:02:01G	6	16.7	1	2.5	0.051
DRB1*13:01:01G	1	2.8	7	17.5	0.058

F—frequency.

## Data Availability

The data that support the findings of this study are available from the corresponding author upon request.

## Supplementary Materials

The following supporting information can be downloaded at: https://www.mdpi.com/article/10.3390/ijms262311638/s1.

## Abbreviations

The following abbreviations are used in this manuscript:

PE	Preeclampsia
OD	Oocyte donation
HLA	Human Leukocyte Antigens
ADCC	Antibody-dependent cellular cytotoxicity
PVCM	Placental villus-conditioned culture medium
PBMC	Peripheral blood mononuclear cells
PBS	Phosphate-buffered saline
ICM	ImmunoCult medium
ROC	Receiver Operating Characteristic
AUC	Area Under the Curve
