# Supplementary material for: Antibody-Dependent Cytotoxicity of Monocytes in Preeclampsia Is Associated with Soluble Forms of HLA
Source: Int J Mol Sci. 2025 Dec 1;26(23):11638. doi: 10.3390/ijms262311638 (PMC12692172; doi:10.3390/ijms262311638)
Supplement: Supplementary file 1 [file ijms-26-11638-s001.zip › Supplementary File S2.pdf]

## Bioinformatic analysis performed to evaluate HLA alleles

We performed two separate Bioinformatic analyses for two predictor variables: maternal HLA alleles (classes I and II) and "paternal" alleles obtained as unique (not matching with maternal) alleles of the child from each mother–child pair. The string names of alleles were unified (clearing spaces; technical replacement of such characters as * and : in column names), after which binary matrices of alleles "individual × allele" presence/absence were formed separately for each panel. The analysis was performed in two groups — Norm and PE; Observations with incomplete data were also included in the respective comparisons. To increase the stability of the estimates, rare characteristics (prevalence <10% in total) were excluded at the pre-filtration stage.

The primary selection of variables was carried out using Fisher's exact test for each allele in the 2x2 model (presence of allele × Norm/PE group) with two-sided hypothesis testing (fisher.test function). For each allele, the absolute and relative frequencies in the groups and the p-value were calculated; the odds ratio (OR) and the 95% confidence interval were also evaluated. The correction for multiple checks was deliberately not applied, since it was considered a search stage; further verification of the signal stability was carried out in the form of multidimensional models.

At the second stage, a “wide” matrix was obtained, where the columns were different HLA, the rows were samples, and then a binary logistic regression was built, including only alleles selected according to the Fisher criterion ($p<0.05$). The dependent variable means belonging to the PE group (PE=1, Norm=0), predictors are binary indicators of selected alleles. For interpretation, the coefficient estimates, their respective OR and 95% confidence intervals, as well as the p-values of the Wald tests were reported. The diagnostic ability of the model was estimated using the ROC curve with the calculation of the area under the curve (AUC) and its 95% confidence interval by the DeLong method. To control overtraining, a stratified k-fold (k=10) cross-validation with averaging of AUC over folds was used; additionally, an error matrix was provided at a probability threshold of 0.5.

All calculations were performed in R (R Foundation) using the packages dplyr, tidyr, purrr, stringr for preprocessing, pROC for ROC analytics (AUC, DeLong method), rpart and rpart.plot for decision trees and their post-pruning, as well as standard tools for generating graphs and exporting tables. The threshold of statistical significance in all tests was set at $\alpha=0.05$; all tests were two-sided.
